# GC/MS-Based Analysis of Fatty Acids and Amino Acids in H460 Cells Treated with Short-Chain and Polyunsaturated Fatty Acids: A Highly Sensitive Approach

**DOI:** 10.3390/nu15102342

**Published:** 2023-05-17

**Authors:** Tianxiao Zhou, Kaige Yang, Yinjie Ma, Jin Huang, Wenchang Fu, Chao Yan, Xinyan Li, Yan Wang

**Affiliations:** School of Pharmacy, Shanghai Jiao Tong University, Shanghai 200240, China; luciferztx@sjtu.edu.cn (T.Z.); ykg19951015@sjtu.edu.cn (K.Y.); bubble1205@sjtu.edu.cn (Y.M.); jinhuang_sjtu@163.com (J.H.); wenchang@sjtu.edu.cn (W.F.); chaoyan@sjtu.edu.cn (C.Y.)

**Keywords:** fatty acids, amino acids, H460 lung cancer cell, GC-MS, targeted metabolomics

## Abstract

The important metabolic characteristics of cancer cells include increased fat production and changes in amino acid metabolism. Based on the category of tumor, tumor cells are capable of synthesizing as much as 95% of saturated and monounsaturated fatty acids through de novo synthesis, even in the presence of sufficient dietary lipid intake. This fat transformation starts early when cell cancerization and further spread along with the tumor cells grow more malignant. In addition, local catabolism of tryptophan, a common feature, can weaken anti-tumor immunity in primary tumor lesions and TDLN. Arginine catabolism is likewise related with the inhibition of anti-tumor immunity. Due to the crucial role of amino acids in tumor growth, increasing tryptophan along with arginine catabolism will promote tumor growth. However, immune cells also require amino acids to expand and distinguish into effector cells that can kill tumor cells. Therefore, it is necessary to have a deeper understanding of the metabolism of amino acids and fatty acids within cells. In this study, we established a method for the simultaneous analysis of 64 metabolites consisting of fatty acids and amino acids, covering biosynthesis of unsaturated fatty acids, aminoacyl-tRNA biosynthesis, and fatty acid biosynthesis using the Agilent GC-MS system. We selected linoleic acid, linolenic acid, sodium acetate, and sodium butyrate to treat H460 cells to validate the current method. The differential metabolites observed in the four fatty acid groups in comparison with the control group indicate the metabolic effects of various fatty acids on H460 cells. These differential metabolites could potentially become biomarkers for the early diagnosis of lung cancer.

## 1. Introduction

Lung cancer is mostly familiar primary malignant tumor of lung [1]. The vast majority of lung cancer originates from the bronchial mucosa epithelium, which is why it is also known as bronchial lung cancer. Lung cancer has the ability to spread throughout the body. Smoking remains the primary cause of lung cancer, yet air pollution and other environmental factors also contribute to an increased risk. Consequently, emphasizing the severity of lung cancer and promoting early screening and preventative measures are crucial in reducing the number of affected individuals and improving the overall quality of life [2].

Fatty acids are the main components of neutral fats, phospholipids, and glycolipids [3,4,5]. When supplied with adequate oxygen, fatty acids can be oxidized and broken down into carbon dioxide and water, releasing a considerable amount of energy [6]. Therefore, fatty acids are one of the primary sources of energy for the body [7]. Fatty acid metabolites participate in the regulation of multiple gene expressions [8]. Recent studies have shown that various cancer cells exhibit alterations in many fatty acid metabolic pathways [9,10,11,12,13,14,15]. During the cancer development, alterations in fatty acid metabolic pathways not only supply energy for the cancer cells but also play an significant role in biomembrane macromolecules and signaling molecules [16]. Fatty acids can be classified into short-chain fatty acids (SCFAs) and polyunsaturated fatty acids (PUFAs). SCFAs are mainly generated by the breakdown of dietary fiber through intestinal microorganisms. In addition, SCFAs penetrate cancer cell nuclei, where they promote histone crotonylation and acetylation, and have the function of inhibition of histone deacetylase activity, thus providing a direct anti-cancer effect because their molecular size is small. PUFAs are a type of fatty acid that include two or more double bonds. Common examples contain omega-3, omega-6, and omega-9 fatty acids, which have many significant physiological functions such as promoting cardiovascular health and reducing inflammation. However, other studies have identified that excessive intake of PUFAs may increase lung cancer risk. In our previous study, the results showed that lecithin cholesterol acyltransferase (LCAT) expression changed with administration [17]. However, there is limited research examining the influence of SCFAs and PUFAs on H460 cells with regards to alterations in amino acid and fatty acid metabolism. Therefore, further investigation is warranted to fully elucidate the metabolic pathways involved.

Metabolomics is a research measure that mimics the concepts of proteomics and genomics, performing quantification of metabolites within living things, and investigating the relevance between metabolites and physiological and pathological alterations [18]. In the human body, these metabolites are converted or consumed through their respective metabolic pathways and participate in physiological processes such as energy supply, substance transport, and maintenance of the internal environment. Common internal metabolites include glucose, lactate, glycerol, free fatty acids, amino acids, urea, etc. [19,20,21]. Amino acids can also be metabolized into nitrogenous compounds such as urea, creatine, etc., and participate in the body’s nitrogen metabolism process [22,23,24]. Endogenous fatty acids are a significant part of human energy sources. They are stored in adipocytes and released through fatty acid metabolic pathways when energy is needed. Fatty acids are one of the essential nutrients in the diet, and a moderate intake of fatty acids can maintain a balance in endogenous fatty acid metabolism. High intake levels of fatty acids may cause excess endogenous fatty acids, while low intake levels of fatty acids may cause fatty acid deficiency, affecting fatty acid metabolism and energy supply. Moreover, different types of free fatty acids in the diet can also have an impact on endogenous fatty acid metabolism. Fatty acid intake can also affect the metabolism of endogenous amino acids. Therefore, it is crucial to conduct a comprehensive metabolomics study on lung cancer treated with different fatty acids using a wide range of amino acids and fatty acids.

To address the aforementioned difficulties, we developed a method for the simultaneous analysis of 65 metabolites that consisted of fatty acid and amino acid biosynthesis, including biosynthesis of unsaturated fatty acids, aminoacyl-tRNA biosynthesis, and fatty acid biosynthesis, by using GC-MS system. Linoleic acid, linolenic acid, sodium acetate, and sodium butyrate were selected to treat H460 cells in order to validate the current method. The results of the metabolomics statistics demonstrated that these four fatty acids had varying effects on the fatty acid and amino acid metabolism of H460 cells. The changes of C20:4, Ala, and Gly may serve as early diagnostic markers for lung cancer.

## 2. Materials and Methods

### 2.1. Materials

Chromatographic grade pyridine, methanol, acetonitrile, and chloroform were purchased from Shanghai Anpel laboratory Technologies Inc., (Shanghai, China). Fatty acid and amino acid standards were purchased from Shanghai Anpel laboratory Technologies Inc., (Shanghai, China). Deionized water was manufactured by a Direct-Q water purification system (Millipore, El Paso, TX, USA). Silylation reagent N,O-Bis(trimethylsilyl)trifluoroacetamide: Trimethylchlorosilane (BSTFA:TMCS) = 99:1, ≥99% was purchased from Shanghai Anpel laboratory Technologies Inc., (Shanghai, China). Metabolites information is shown in Table S1.

### 2.2. Cultured Cells

H460 cells were acquired from Xinheng Biotechnology Co., Ltd., (Guangzhou, China) and cultured in 1640 medium with added 10% FBS and 1% P/S. Cell culture dishes were placed in an incubator with 5% CO_2_, constant temperature of 37 °C, and saturated humidity. The cells were also treated with the complete medium. The medium was replaced with fresh medium in a timely manner based on cell growth speed. Prior to sample pretreatment, the cells were treated with 500 μM of four fatty acids, respectively, for 24 h. Specifically, the four kinds of fatty acids were sodium acetate, sodium butyrate, linoleic acid, and linolenic acid.

### 2.3. Calibrators and Quality Control Samples

Stock solution: The amino acid and fatty acid standards were dissolved in chloroform to create concentrations of 1 mg/mL solutions, which were then diluted with chloroform to make standard solutions.

Mixed standard solution: Stock solutions and internal standards were correctly measured to create a mixed standard solution with a concentration of 10× (10 ng/μL). The 10× mixed standard solution was then diluted to 3×, 1×, 0.7×, 0.5×, 0.3×, 0.1×, 0.07×, 0.05×, 0.03×, 0.01×, and 0.007× to create a standard linear working solution.

### 2.4. Method Validation

Accuracy and precision were determined on the same day and for 3 consecutive days (n = 6).

In this research, the 0.03×, 0.3×, and 1× samples were chosen for validation of recovery.

### 2.5. GC-MS Sample Preparation

H460 cells grown in log phase were obtained, and the cells were washed three times with PBS after the medium was aspirated. In total, 0.5 mL methanol was added at 4 °C, using a cell scraper to scrape down cells, and then transferred to a 2 mL centrifuge tube. After adding same concentration of internal standard to each sample, grinding beads were added to homogenize each sample by using a cell homogenizer, 300 Hz, 60 s, for three cycles. A total of 250 μL water and chloroform were added afterwards and homogenized again for three cycles. This was then centrifuged at 10,000× *g* for 12 min at 4 °C, then the supernatant was collected using a nitrogen blowing instrument to spin-dry, and then finally reconstituted with 400 μL water/acetonitrile (v:v = 1:1) solution. A total of 200 μL was removed and added into the injection liner, where we continued to spin it dry. The derivatization experiment was then performed. A total of 80 μL pyridine and 80 μL BSTFA was added to the spin-dried liner and incubated at 70 °C for 30 min. The derived samples were then loaded into GC-MS.

### 2.6. GC-MS Analysis

Analysis was performed by 7890B/5977 gas chromatograph-mass spectrometer (Agilent Technologies, Santa Clara, CA, USA) with a HB-5 capillary column (30 m × 0.25 mm × 0.25 µm film thickness) (Agilent Technologies, Santa Clara, CA, USA). The injector temperature was 270 °C, ion source temperature was 230 °C, quadrupole temperature was 150 °C, and interface temperature was 280 °C. The carrier gas was helium and the flow rate was kept at 1 mL/min. The injection volume was 10 µL. The solvent delay time was 3.5 min. The split ratio was 10:1. The gradient heating program of the column temperature box was as follows: the initial temperature was 100 °C, increased to 200 °C at a rate of 2 °C/min and held for 32 min, and finally increased to 280 °C at a rate of 10 °C/min and kept for 35 min.

The ion source was electron impact (EI) mode and the voltage was 70 eV. Compounds were qualified and quantified with selected ion monitoring (SIM) mode by utilizing characteristic ions. The SIM parameters of metabolites are in Table S2.

### 2.7. Statistical Analysis

The collected raw data were obtained from Masshunter 12.0 (Agilent Technologies, Santa Clara, CA, USA), and the concentration information of the metabolites were retrieved from the standard curve.

Statistical analyses were carried by utilizing Simca 14.1 (Sartorius Company, Goettingen, Germany) and GraphPad Prism 8.0 (GraphPad Software Inc., San Diego, CA, USA). PCA and OPLS-DA are used to survey the diversities between the groups. In order to verify the stability and prediction accuracy of the model, cyclic interactive verification and response sequencing tests were concurrently carried out in this process to prevent the model from overfitting. VIP > 1 was contemplated as a variance variable in multi-dimensional statistics. In order to further screen, we continued to process the single dimension data statistically, using Students’ *t*-test and the multiple change analysis. Ultimately we screened metabolites with ANOVA *p*-value < 0.05, maximum CV < 30% and fold-change (FC) > 1.5 or <1/1.5 as the difference variable for further analysis. In order to better understand the correlation of differential metabolites, a volcano plot, heatmap, network analysis, and correlation analysis were utilized to search for potential biomarkers of lung cancer with high sensitivity and specificity through MetaboAnalyst 4.0 (Xia Lab @ McGill, Ste. Anne de Bellevue, QC, Canada). For all experiments, statistical significance was defined as * *p* < 0.05 and ** *p* < 0.01.

## 3. Results

### 3.1. Pretreatment Optimization

The choice of redissolved solvent before derivatization is critical in GC-MS sample pretreatment as it directly impacts the yield of the derivatization reaction and thus affects the response strength of mass spectrometry. We investigated several solvents, including methanol, acetonitrile, ethyl acetate, toluene, pyridine, and acetone. As shown in Figure S1, the derivatization with pyridine and acetone had the highest response after redissolution, while the response of other solvents was lower than that of pyridine and acetone. However, considering the similar re-dissolution effect of pyridine and acetone and the greater reproductive toxicity of pyridine compared to acetone, we selected acetone as the solvent for redissolution in this study.

### 3.2. GC-MS Method Optimization

In gas chromatography, optimizing the inlet temperature and temperature program is crucial as these parameters determine the sample analysis time, the resolution of target compounds, and the collection sensitivity. Initially, we optimized the inlet temperature. As illustrated in Figure S2, the target response significantly improved when the temperature was increased from 260 °C to 270 °C. However, when the temperature increased from 270 °C to 280 °C, the target response decreased. Therefore, we selected 270 °C as the inlet temperature for this study.

**Figure 1:**
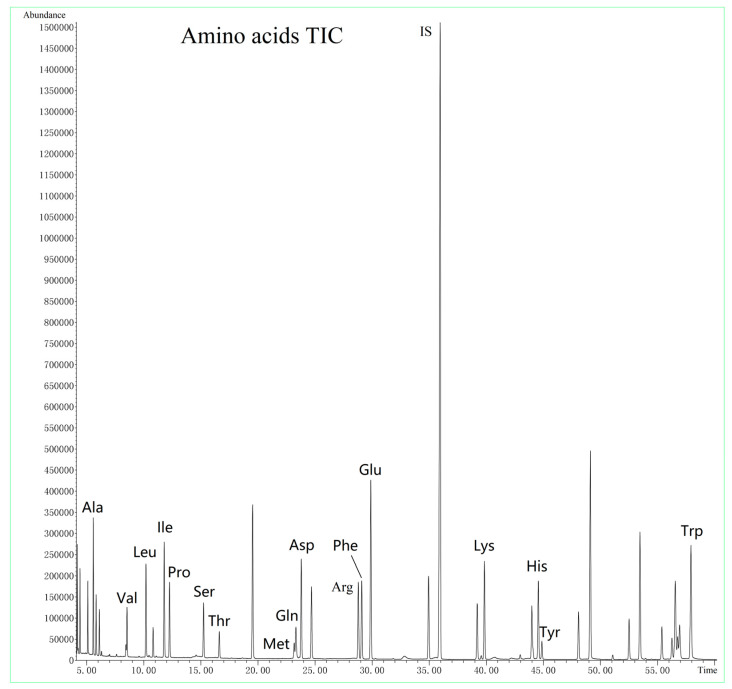
Total ion chromatograms of the amino acids mixture.

**Figure 2:**
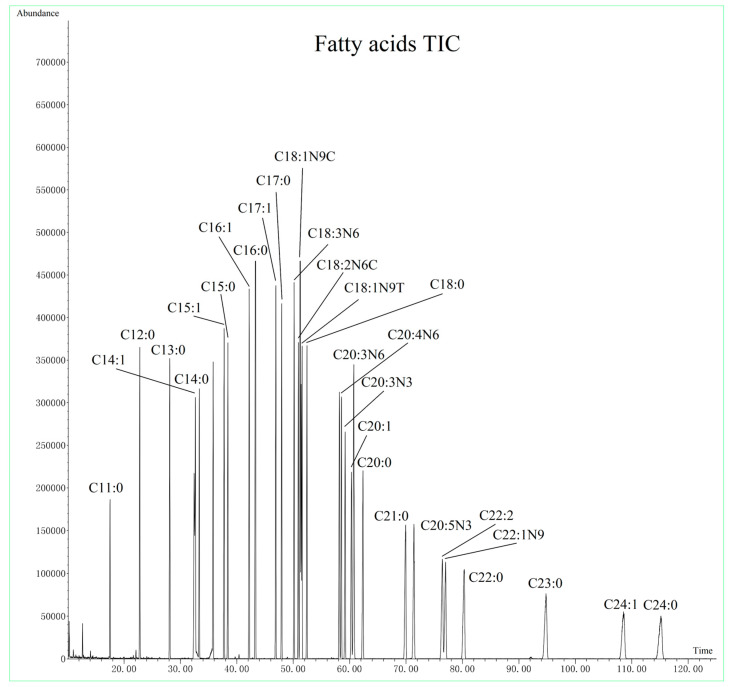
Total ion chromatograms of the fatty acids mixture.

Next, we examined the effect of different temperature gradients on target analysis. Temperature gradient mainly affects retention time and resolution. After optimization, we were able to slightly reduce the sample running time without compromising the separation between the targets. The total ion chromatogram (TIC) of amino acids mixture is displayed in Figure 1 and the TIC of the fatty acids mixture is illustrated in Figure 2.

### 3.3. Methodology Validation

The validation results of the method are presented in Table S3. The LODs ranged from 0.033 to 100.00 ng/μL, while the LOQs ranged from 0.10 to 300.00 ng/μL. The majority of metabolites exhibited excellent linearity, as indicated by R^2^ values exceeding 0.99. Furthermore, the CV% of the majority of the compounds were less than 20%, demonstrating the high precision of this method.

### 3.4. CCK8 Experiment

In the study of cell metabolomics, the first step is to conduct a cell proliferation experiment on all groups. This is to make sure that the dosage has no impact on cellular growth and to enable further analysis of metabolic changes. To achieve this, we carried CCK8 experiment on all groups. The results were placed in our previous study [17]. When the concentration of all fatty acids was 500 μM, the cell proliferation was not significantly impacted. Yet, when the concentration of linolenic acid continued to rise, the cell proliferation was influenced to varying degrees. Therefore, we selected 500 μM for all fatty acid groups.

### 3.5. Statistical Analysis of Targeted Metabolomics

Due to the metabolite concentration as the final variable directly reflects the content of an individual metabolite in an individual sample and the response intensity of the variable is not on a uniformity standard, it greatly affects the statistical results during processing. Thus, we used Pareto scaling to increase each variable’s comparability in various samples. This ensures that noise interference is not amplified while also taking into account the contributions of both low and high response intensity metabolites. Please refer to Figure S3 for the discrepancies in comparison before standardization and after standardization.

**Figure 3:**
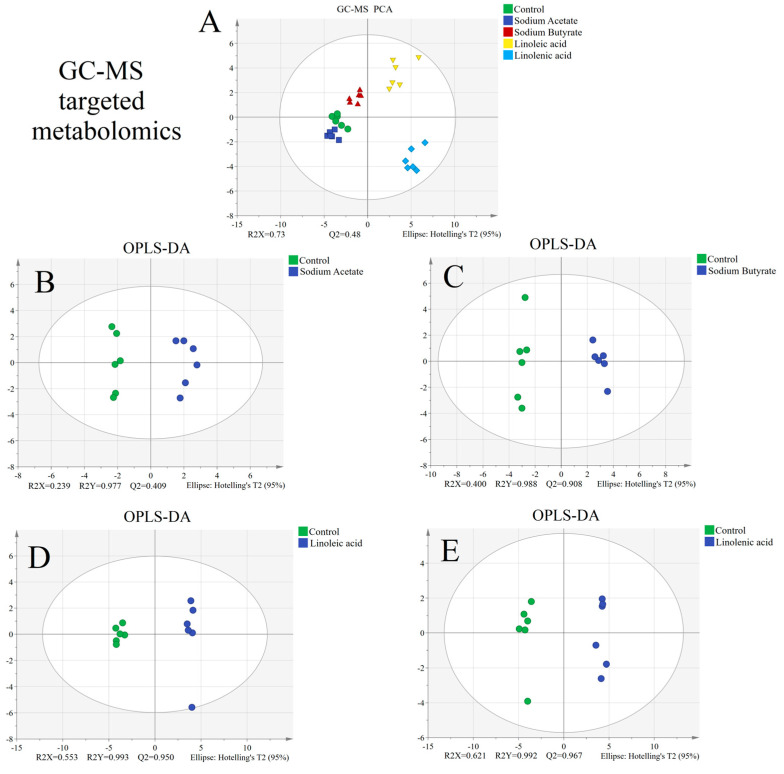
Statistical results between the control group and four fatty acid groups: (**A**) PCA plots, (**B**–**E**) OPLS−DA plots of all four fatty acid groups compared with the control group.

SIMCA software was used for statistical analysis. The PCA results showed that the whole groups were clustered within 95% confidence intervals (see Figure 3A), indicating that extreme outliers did not need to be excluded. This result exhibited preferable repeatability because of its fine intra-group aggregation. From the results, we screened that the sodium acetate group was closely clustered with the control group, while the other groups showed good separation. To further investigate the differences between the fatty acid group and the control group, we carried on OPLS-DA analysis.

OPLS-DA analyses were employed to investigate differences between the dose group and the control group. The OPLS-DA results (Figure 3B–E) showed complete separation between the two groups. The model parameters such as R2X, R2Y, and Q2 (Figure 3B–E) indicated this model had prediction abilities and good fitting. Analysis results indicated remarkable differences between all four fatty acid groups and the control group. To identify the differential metabolites among the groups, we subsequently conducted drawing and analysis of the volcano plots.

**Figure 4:**
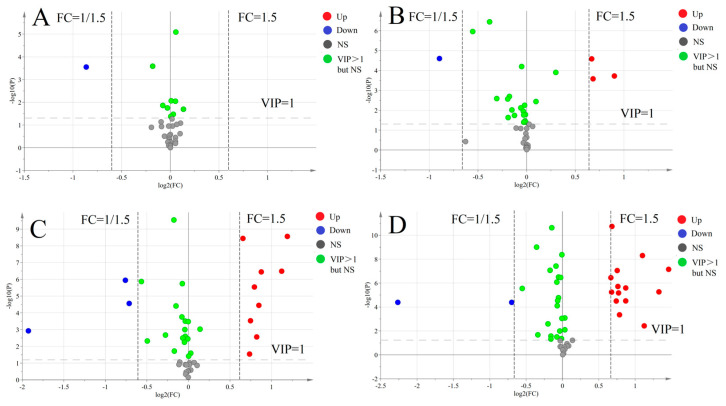
Volcano plots between the control group and four fatty acids group: (**A**–**D**) volcano plots of four fatty acid groups compared with the control group.

The volcano plots for targeted metabolism are shown in Figure 4A–D. Based on the analysis results, we selected metabolites with *p*-value < 0.05, VIP > 1.0 and FC > 1.5 or <1/1.5. By examining the volcano plots, we identified the metabolites that differed significantly between control groups and four fatty acid groups and conducted follow-up analysis. We created heatmaps and a correlation heatmap of the selected differential metabolites.

**Figure 5:**
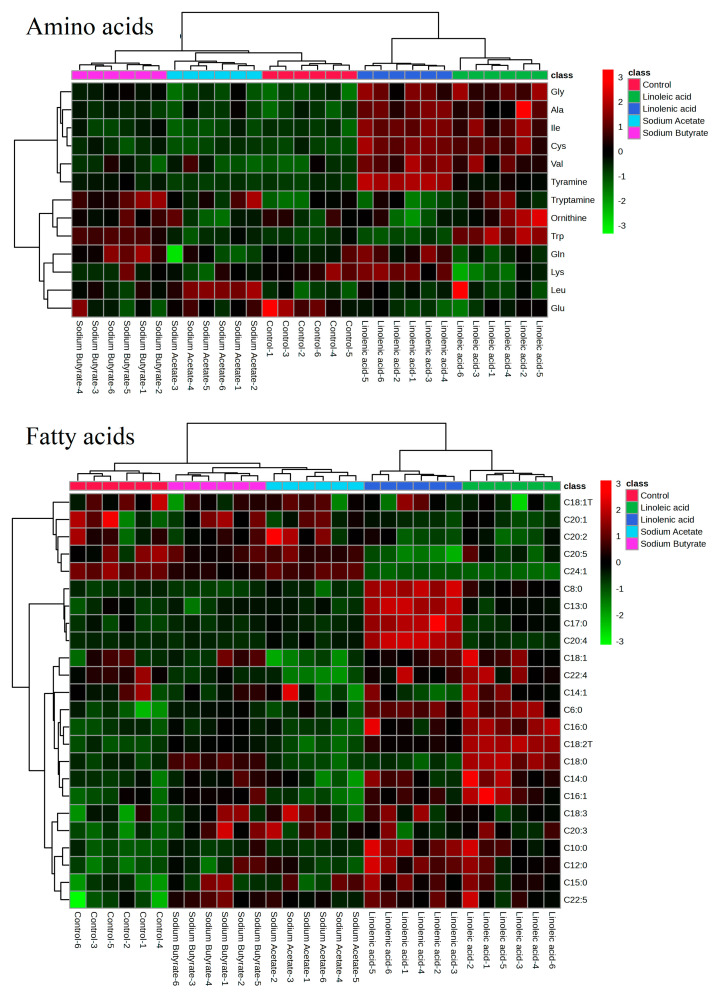
Heatmaps based on targeted metabolomics.

The heatmaps in Figure 5 provide a clear visualization of the differential regulation of target compounds in response to different treatments. These variations in the compound profiles are crucial in understanding the underlying mechanisms and potential biological effects associated with each treatment.

The SCFA group demonstrated a distinct pattern of regulation compared to the control group. The down-regulation of C22:4 in the SCFA group could be indicative of reduced elongation or desaturation activity in the lipid metabolism pathway. This observation may also suggest that SCFA treatment influences the balance of fatty acids in the system, promoting the generation of other fatty acid species. The up-regulation of C20:5 and C20:2 in the SCFA group, on the other hand, could be linked to increased biosynthesis or reduced degradation of these fatty acids, potentially leading to altered cellular functions or signaling pathways.

In contrast, the PUFA group exhibited an up-regulation of C22:4, which could be a result of increased elongation or desaturation of precursor fatty acids or reduced degradation. The down-regulation of C20:5 and C20:2 in the PUFA group might suggest a shift in the balance of fatty acids towards the production of other species, possibly due to the influence of the PUFA treatment on lipid metabolism.

The heatmap analysis presented in Figure 5 highlights the intricate relationship between different treatments and the regulation of target compounds. These findings have implications for understanding the potential physiological effects of SCFA and PUFA treatments and could serve as a basis for future research to explore their potential therapeutic applications. Further investigations are warranted to elucidate the precise mechanisms underlying the observed changes in compound profiles and to determine the potential benefits or drawbacks of manipulating these fatty acids in different biological contexts.

**Figure 6:**
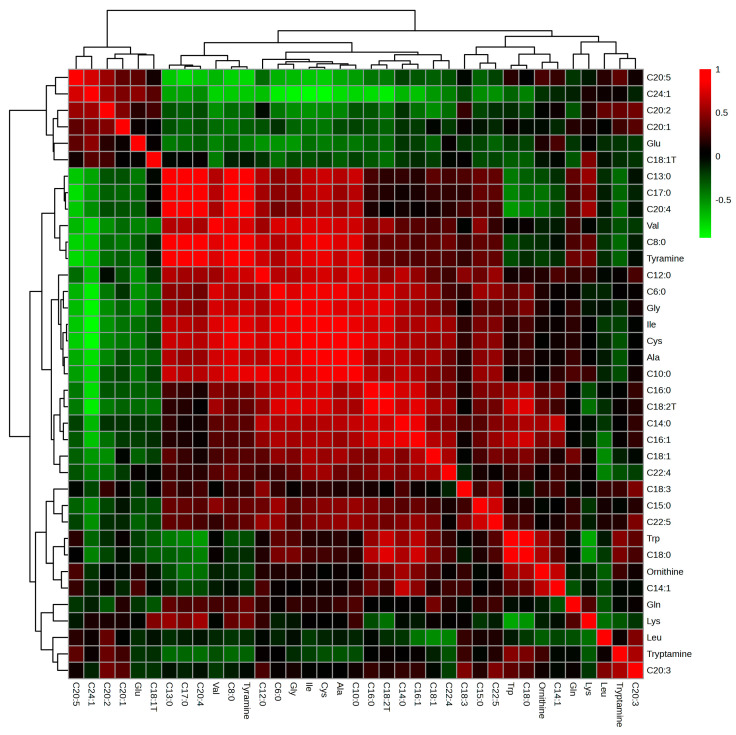
Correlation analysis based on targeted metabolomics.

Figure 6 provides insights into the relationships between various metabolites, revealing both positive and negative correlations among them. These correlations offer valuable information regarding the metabolic pathways and potential interactions between the compounds which can help further understand the biological processes and effects of various treatments.

The positive correlation between C20:5 and C24:1 suggests that these two fatty acids might share common biosynthetic pathways or regulatory mechanisms. Conversely, the negative correlation between C20:5 and C20:4 implies a potential competition between these two fatty acids for the same precursor molecules or enzymes, which could result in a trade-off between their biosynthesis.

Similarly, the positive correlation between Leu (leucine) and C20:3 indicates that they might be influenced by the same metabolic pathways or regulatory factors. The negative correlation between Leu and C22:4, on the other hand, could reflect competition for precursors or regulatory elements that control their biosynthesis or degradation.

The positive correlation between Lys (lysine) and C20:4 implies a possible link between their biosynthesis or regulation, while the negative correlation with Trp (tryptophan) may suggest competition for shared resources in their metabolic pathways.

C8:0, a medium-chain fatty acid, was positively correlated with C20:4, which might point to shared metabolic pathways or regulatory mechanisms. The negative correlation between C8:0 and C20:5 could indicate competition between the two fatty acids for the same precursor molecules or enzymes.

Lastly, Ala (alanine) and Val (valine) display a positive correlation, which could be a result of similar biosynthesis or regulatory processes. The negative correlation between Ala and C24:1 suggests potential competition for precursors or regulatory elements involved in their metabolism.

**Figure 7:**
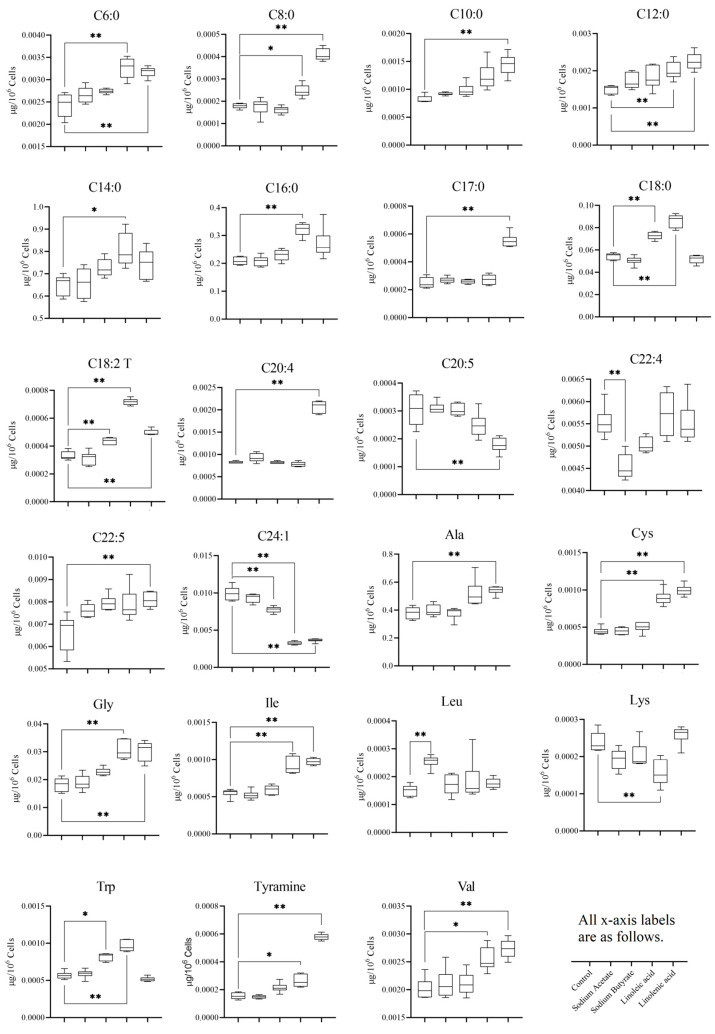
Boxplots of differential metabolites that contained fatty acids and amino acids. For all experiments, statistical significance was defined as * *p* < 0.05 and ** *p* < 0.01.

Figure 7 presents the boxplots of metabolite levels, providing a clear visualization of the distribution and variability of each metabolite across different treatment groups. When comparing these results with the previously mentioned volcano plot findings, it becomes evident that the PUFA group exhibited a higher number of differential metabolites than the SCFA group. This observation suggests that the PUFA treatment may have a more pronounced impact on the metabolic profile, potentially due to its stronger influence on multiple biological pathways or regulatory mechanisms.

Among the identified differential metabolites, C20:4 (arachidonic acid), Ala (alanine), and Gly (glycine) stand out as potential biomarkers for lung cancer. The altered levels of these metabolites in the context of lung cancer could be indicative of disrupted metabolic processes or signaling pathways associated with the disease. For instance, C20:4 is a precursor for the synthesis of various eicosanoids, which are bioactive lipid mediators involved in the inflammation and immune response. Dysregulation of C20:4 metabolism could, therefore, contribute to chronic inflammation and cancer development.

Ala and Gly, both non-essential amino acids, may also be linked to lung cancer through alterations in amino acid metabolism or their involvement in other biological processes. For example, Gly is a precursor for the synthesis of proteins, nucleic acids, and glutathione, a crucial antioxidant that protects cells from oxidative stress. Disruptions in Gly metabolism could impair cellular defense mechanisms and promote cancer development.

The identification of these potential biomarkers offers valuable information for the early detection and monitoring of lung cancer. Moreover, it provides a starting point for further research into the underlying mechanisms connecting these metabolites to lung cancer development and progression. Future studies should focus on validating the diagnostic potential of these biomarkers in larger, independent cohorts, as well as investigating their roles in cancer biology. Additionally, understanding how PUFA and SCFA treatments impact these potential biomarkers may offer insights into novel therapeutic strategies for lung cancer.

## 4. Discussion

Lung cancer, especially non-small cell lung cancer (NSCLC), is one of most prevalent kinds of cancer. Consequently, lung cancer’s mortality rate remains very high, making it the main cause of cancer deaths in the world.

The proliferation of cancer cells requires the presence of fatty acids and phospholipids [25]. Platelet-activating factor (PAF) is an effective phospholipid that can facilitate the proliferation and metastasis of lung cancer. Phospholipase A2 hydrolyzes PAF to yield lysophospholipid PAFs. (lysoPAFs) [26]. The level and function of phospholipase A2 increases in lung cancer patients. Enzymes facilitate the process of oxidizing phospholipids, resulting in the formation of lysoPAFs and free fatty acids (FFAs). These compounds are known to significantly contribute to the development, progression, and metastasis of tumors, as well as promoting angiogenesis and lymphangiogenesis. LysoPAFs (such as lysophosphatidylcholine) have been considered potential biomarkers of ovarian cancer. Phospholipids found in the plasma, such as lysophosphatidylcholine and phosphatidylcholine, have the potential to serve as biomarkers for prostate cancer and lung cancer.

Animals with a high dietary intake of linoleic acid have been observed to exhibit an increased risk of breast cancer metastasis, which provides support for the theory that elevated levels of free fatty acids may promote cancer [27]. Hydroxyeicosatetraenoic acid (HETE) and hydroxyoctadecadienoic acid (HODES) are steady oxidation products of arachidonic acid (AA). Cell culture, tissue, and animal models have yielded compelling evidence that FFA and oxidized FFA HETE and HODE play a significant role in the development, progression, and metastasis of lung cancer. Although the exact role of HODE is still unclear, it has been associated with many cancers. Studies have identified 9-HODE and 13-MODE as peroxisome proliferator-activated receptor gamma ligands, which have been implicated in promoting the progression and metastasis of lung cancer.

Amino acids are fundamental components that make up biological proteins and are closely linked to life processes [28,29]. Amino acids have specific physiological functions in antibodies and are essential nutrients in organisms. Glutamine is an essential nutrient for the majority of cancer cells grown in vitro as it can serve as a source of carbon to make up for the deficit of the TCA cycle in cancer cells [30]. However, the extent of glutamine metabolism in vivo remains a subject of debate and there is evidence indicating that certain tumors undergo net glutamine synthesis. Analysis of tumor metabolism in lung cancer patients suggests that glucose and glutamine may be essential for TCA cycle function. As potential cancer markers, the study of amino acids and fatty acids in lung cancer are urgently needed.

**Figure 8:**
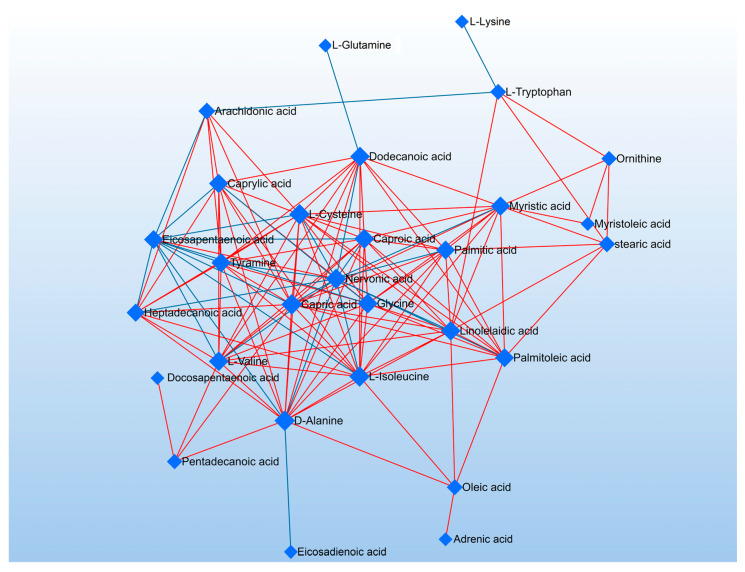
Network analysis of differential metabolites in targeted metabolomics.

Based on the statistical results presented above, it is evident that the impact of four distinct fatty acid treatments on cell metabolism demonstrated a noteworthy interference. In particular, the sodium acetate and butyrate groups were found to have identified 1 and 4 distinct metabolites, respectively, while the linoleic acid and linolenic acid groups showed 12 and 16 different metabolites, respectively. Furthermore, there were notable changes in fatty acids and amino acids. We then conducted OPLS-DA analysis for the SCFA and PUFA groups. As depicted in Figure S4, there is a noteworthy contrast between the SCFA group and the PUFA group. Changes in metabolites were found to be associated with several metabolic pathways, which may uncover various potential mechanisms of administering fatty acids and their metabolites. Based on the KEGG database, some pathways, such as the biosynthesis of unsaturated fatty acids, aminoacyl-tRNA biosynthesis, and fatty acid biosynthesis, were found to be involved in the four fatty acid treatments. Notably, the polyunsaturated fatty acid group exhibited significant metabolic alterations in both the linoleic and linolenic acid groups. As in Figure 8, the network analysis indicated that metabolites such as Gly, C24:1, and Tyr are located in the central position of the network. From the perspective of network density, metabolites such as Tyr, C10:0, and Cys have higher network density, indicating a tighter interaction and more complex metabolic pathways among them. Furthermore, as shown in Figure 7, C20:4, Ala, and Gly among the differential metabolites may become potential biomarkers of lung cancer.

Persistent inflammation is commonly characterized by the buildup of various components including inflammatory cells, growth factors, cytokines, oxidants, and pro-inflammatory lipid mediators. These elements are believed to act together in a synergistic manner to facilitate the creation and sustenance of an activated matrix, as well as the growth and division of epithelial cells [31]. The process of wound healing is closely linked to both inflammation and cellular proliferation, which are essential for tissue regeneration. In cases where inflammation becomes excessive, proliferating cells may acquire genetic alterations that enable them to sustain growth under these conditions [10]. When tissues are damaged, undergo repair, or develop cancer, arachidonic acid and linoleic acid undergo increased oxidation rates via the cyclooxygenase and lipoxygenase pathways, producing potent lipid molecules called eicosanoids. These eicosanoids, derived from arachidonic acid or hydroxy-fatty acids derived from linoleic acid, play critical roles in fundamental biological processes such as cell growth, cell survival, angiogenesis, cell invasion, metastasis, and immune regulation. However, the oxidation reaction of long-chain unsaturated fatty acids shows that different lipoxygenase subtypes have antipodal function during carcinogenesis; specifically, type 5 and platelet type 12 lipoxygenase have carcinogenic activity, while 15-lipoxygenase-1 and 15-lipoxygenase-2 may inhibit carcinogenesis.

Patients’ weight loss with lung cancer is related to impaired treatment outcomes and reduced survival. The weight loss experienced by lung cancer patients is characterized by the depletion of fat mass and skeletal muscle, while internal organs are usually preserved or may even enlarge. Although it has been reported that cancer patients experience profound changes in host substrate metabolism, the mechanism underlying weight loss in these patients remains unclear. Isotopic tracer studies have shown that protein breakdown and glucose turnover are increased in patients with lung cancer. Increased alanine gluconeogenesis has been observed in liver cells affected by tumors, tumor-bearing animals in vivo, and cancer patients of various tumor types [28]. Recently, we reported an increase in systemic alanine gluconeogenesis in lung cancer patients with weight loss. We also found a significant correlation between alanine gluconeogenesis and weight loss. In a recent study using 31P MRS, different concentrations of PME were detected in the livers of cancer patients with varying tumor types, including lung cancer patients. In contrast, the liver PME levels of cancer patients with stable weight were not significantly dissimilar to that of individuals who were in good health. Furthermore, in lung cancer patients, the liver PME level was significantly correlated with the alanine gluconeogenesis rate, but not in healthy subjects. MRS research was also used to obtain dynamic information on liver metabolism by monitoring changes in liver metabolite concentration during the infusion of gluconeogenic substrate [29]. Studies using 31P MRS and L-alanine infusion in healthy rats, ischemic rats, or postoperative rats have reported changes in PME and ATP levels. In healthy individuals, 31P MRS and a dose or continuous infusion of L-alanine have been shown to offer data on the concentration changes of gluconeogenic intermediates in liver.

Identification of key factors that contribute to tumor initiation cell (TIC) status may open up new paths for the treatment of cancer. Glycine decarboxylase (GLDC), a metabolic enzyme, is essential for TICs in NSCLC [2]. NSCLC tumors exhibit heightened expression levels of the stem cell factors LIN28B and GLDC, both of which are essential for the growth and tumorigenesis of TICs. Interestingly, GLDC and other glycine or serine enzymes’ overexpression, rather than their inactivation, facilitates cell transformation and tumorigenesis. GLDC provokes significant alterations in glycolysis and glycine/serine metabolism, resulting in changes in pyrimidine metabolism and the regulation of cancer cell proliferation. In clinical scenarios, abnormal activation of GLDC has been linked to unfavorable survival outcomes in lung cancer patients, and anomalous GLDC expression has been detected in various cancer types. Therefore, targeting the connection between glycine metabolism and tumorigenesis may offer novel strategies for promoting anticancer therapy.

## 5. Conclusions

We utilized a metabolomics-based strategy for detecting alterations in metabolism caused by the administration of sodium acetate, sodium butyrate, linoleic acid, and linolenic acid in H460 cells. The results demonstrated that exposure to the four fatty acids resulted in notable alterations in the metabolic profile of H460 cells as compared to the control group. Alterations in the metabolic landscape require the involvement of various metabolic pathways, including the biosynthesis of unsaturated fatty acids, aminoacyl-tRNA biosynthesis, and fatty acid biosynthesis. Furthermore, the H460 cells responded differently to each metabolite, indicating unique changes in response to the dose of the fatty acid. For example, the administration of sodium butyrate resulted in a more pronounced disruption of the cellular metabolic profile compared to sodium acetate, whereas the effect of both sodium acetate and sodium butyrate was weaker than that of linoleic acid and linolenic acid. Finally, our findings indicate that metabolomics has proven to be a potent means of evaluating health status and generating information for a comprehensive assessment of health risks associated with the consumption of fatty acids.

## Data Availability

All relevant data are included in the article.

## Supplementary Materials

The following supporting information can be downloaded at: https://www.mdpi.com/article/10.3390/nu15102342/s1, Table S1. Target compound information. Table S2. Target compound GC-MS parameters. Table S3. LOD, LOQ, linear range and methodology validation data of metabolites. Figure S1. Comparison of the effect of different solvents on redissolution. Figure S2. Comparison of different inlet temperatures. Figure S3. Data of targeted metabolism were compared before and after normalization. Figure S4. OPLS-DA plots between short-chain fatty acids and polyunsaturated fatty acids.
