# Supplementary material for: GC/MS-Based Analysis of Fatty Acids and Amino Acids in H460 Cells Treated with Short-Chain and Polyunsaturated Fatty Acids: A Highly Sensitive Approach
Source: Nutrients. 2023 May 17;15(10):2342. doi: 10.3390/nu15102342 (PMC10220902; doi:10.3390/nu15102342)
Supplement: Supplementary file 1 [file nutrients-15-02342-s001.zip › nutrients-2383904-supplementary.pdf]

GC/MS-based analysis of fatty acids and amino acids in H460 cells treated with short-chain and polyunsaturated fatty acids: a highly sensitive approach

Tianxiao Zhou, Kaige Yang, Yinjie Ma, Jin Huang, Wenchang Fu, Chao Yan, Xinyan Li * and Yan Wang *

School of Pharmacy, Shanghai Jiao Tong University, Shanghai 200240, China
* Correspondence: lixy@sjtu.edu.cn (X.L.); wangyan11@sjtu.edu.cn (Y.W.); Tel.: +86-21-3420-4772 (X.L.); +86-21-3420-5673 (Y.W.).

Table S1 Target compound information

No.	Analytes	Formula	CAS No.	Common Name
1	Ala	C3H7NO2	338-69-2	D-Alanine
2	Val	C5H11NO2	72-18-4	L-Valine
3	Leu	C6H13NO2	61-90-5	L-Leucine
4	Ile	C6H13NO2	73-32-5	L-Isoleucine
5	Pro	C5H9NO2	147-85-3	L-Proline
6	Gly	C2H5NO2	56-40-6	Glycine
7	Ser	C3H7NO3	56-45-1	L-Serine
8	Thr	C4H9NO3	72-19-5	L-Threonine
9	Asp	C4H7NO4	56-84-8	L-Aspartic acid
10	Met	C5H11NO2S	63-68-3	L-Methionine
11	Cys	C3H7NO2S	52-90-4	L-Cysteine
12	Arg	C6H14N4O2	74-79-3	L-Arginine
13	Glu	C5H9NO4	56-86-0	L-Glutamic acid
14	Phe	C9H11NO2	63-91-2	L-Phenylalanine
15	Asn	C4H8N2O3	70-47-3	L-Asparagine
16	Gln	C5H10N2O3	56-85-9	L-Glutamine
17	His	C6H9N3O2	71-00-1	L-Histidine
18	Lys	C6H14N2O2	56-87-1	L-Lysine
19	Tyr	C9H11NO3	60-18-4	L-Tyrosine
20	Trp	C11H12N2O2	73-22-3	L-Tryptophan
21	Ornithine	C5H12N2O2	3184-13-2	Ornithine
22	Dihydroxyphenylacetic acid	C8H8O4	102-32-9	3,4-Dihydroxybenzeneacetic acid
23	Tyramine	C8H11NO	51-67-2	Tyramine
24	Dopamine	C8H11NO2	62-31-7	Dopamine
25	Tryptamine	C10H12N2	61-54-1	Tryptamine
26	Quinolinic acid	C7H5NO4	89-00-9	Quinolinic acid
27	3-Hydroxyanthranilic acid	C7H7NO3	548-93-6	3-Hydroxyanthranilic acid
28	C6:0	C6H12O2	142-62-1	Caproic acid
29	C8:0	C8H16O2	124-07-2	Caprylic acid

30	C10:0	C10H20O2	334-48-5	Capric acid
31	C11:0	C11H22O2	112-37-8	Undecanoic acid
32	C12:0	C12H24O2	143-07-7	Dodecanoic acid
33	C13:0	C13H26O2	638-53-9	Tridecanoic acid
34	C14:1	C14H26O2	544-64-9	Myristoleic acid
35	C14:0	C14H28O2	544-63-8	Myristic acid
36	C15:1	C15H28O2	26444-04-2	Pentadecenoic acid
37	C15:0	C15H30O2	1002-84-2	Pentadecanoic acid
38	C16:1	C16H30O2	373-49-9	Palmitoleic acid
39	C16:0	C16H32O2	57-10-3	Palmitic acid
40	C17:1	C17H32O2	1981-50-6	9Z-Heptadecenoic acid
41	C17:0	C17H34O2	506-12-7	Heptadecanoic acid
42	C18:3	C18H30O2	463-40-1	alpha-Linolenic acid
43	C18:2T	C18H32O2	506-21-8	Linolelaidic acid
44	C18:1T	C18H34O2	693-72-1	Vaccenic acid
45	C18:2	C18H32O2	60-33-3	Linoleic acid
46	C18:1	C18H34O2	112-80-1	Oleic acid
47	C18:0	C18H36O2	57-11-4	stearic acid
48	C20:4	C20H32O2	506-32-1	Arachidonic acid
49	C20:5	C20H30O2	10417-94-4	Eicosapentaenoic acid
50	C20:3C	C20H34O2	17046-59-2	Eicosatrienoic acid
51	C20:2	C20H36O2	5598-38-9	Eicosadienoic acid
52	C20:1	C20H38O2	5561-99-9	11Z-Eicosenoic acid
53	C20:3	C20H34O2	1783-84-2	Dihomo-gamma-linolenic acid
54	C20:0	C20H40O2	506-30-9	Arachidic acid
55	C21:0	C21H42O2	2363-71-5	Heneicosanoic acid
56	C22:6	C22H32O2	6217-54-5	Docosahexaenoic acid
57	C22:4	C22H36O2	28874-58-0	Adrenic acid
58	C22:5	C22H34O2	24880-45-3	Docosapentaenoic acid
59	C22:2	C22H40O2	17735-98-7	Docosadienoate (22:2n6)
60	C22:1	C22H42O2	1002-96-6	Cetoleic acid
61	C22:0	C22H44O2	112-85-6	Behenic acid
62	C23:0	C23H46O2	2433-96-7	Tricosanoic acid
63	C24:1	C24H46O2	506-37-6	Nervonic acid
64	C24:0	C24H48O2	557-59-5	Tetracosanoic acid
65	2-chloro-3-phenyl-DL-alanine	C9H10ClNO2	14091-11-3	2-amino-3-(2-chloro-phenyl)-propionic acid

Table S2 Target compound GC-MS parameters

Compound	Retention time	Characteristic ions
C8:0	11.08	158
C10:0	12.64	186
C11:0	17.51	200
C12:0	22.78	214
C13:0	28.10	228

C14:0	33.34	242
C14:1	32.65	208
C15:0	38.40	256
C15:1	37.75	222
C16:0	43.33	270
C16:1	42.19	236
C17:0	47.95	284
C17:1	46.91	250
C18:0	52.44	298
C18:1N9T	51.57	296
C18:1N9C	51.25	296
C18:2N6T	51.40	294
C18:2N6C	50.92	294
C18:3N6	50.16	292
C20:0	62.37	326
C18:3N3	60.30	292
C20:1	60.75	292,324
C21:0	69.90	340
C20:2	60.32	322
C20:3N6	60.78	320
C22:0	80.32	354
C20:3N3	59.20	320
C20:4N6	58.13	318
C22:1N9	77.02	352
C23:0	94.80	368
C22:2	76.43	350
C20:5N3	71.41	79,91
C24:0	115.20	382
C24:1	108.54	348,380
C22:6N3	69.92	342
Ala	5.23	116
Val	8.56	144
Leu	10.90	158
Ile	11.88	158
Pro	11.94	142
Gly	12.30	174
Ser	15.36	204
Thr	16.74	117
Asp	23.99	232
Met	23.23	176
Cys	25.50	220
Arg	28.68	142
Glu	29.22	246
Phe	29.00	218

Asn	31.90	116
Gln	23.40	156
His	45.00	254
Lys	40.58	174
Tyr	45.00	218
Trp	57.40	202
2-cl-Phe(IS)	35.84	218

Table S3 LOD, LOQ, linear range and methodology validation data of metabolites.

Compound	Linear range/μg*mL^{-1}	LOD/μg*mL^{-1}	LOQ/μg*mL^{-1}	LQC/0.03X			MQC/0.3X			HQC/1X		
				RE %	CV% Intra(n=6)	CV% Inter(n=6)	RE %	CV% Intra(n=6)	CV% Inter(n=6)	RE %	CV% Intra(n=6)	CV% Inter(n=6)
Ala	0.033-23.33	0.033	0.10	6.46	12.46	9.59	2.00	5.52	2.41	6.42	2.24	3.82
Val	0.033-23.33	0.033	0.10	6.91	9.82	9.38	8.94	8.36	6.70	8.80	3.95	4.45
Leu	0.033-23.33	0.033	0.10	11.22	6.55	13.95	13.78	8.17	7.31	10.28	6.59	1.73
Ile	0.033-23.33	0.033	0.10	14.76	7.64	8.53	11.95	7.37	7.74	8.31	2.32	7.94
Pro	0.033-23.33	0.033	0.10	11.07	9.36	11.04	7.06	11.44	13.34	9.90	1.42	0.16
Gly	0.033-23.33	0.033	0.10	14.06	7.79	13.19	6.71	13.43	13.58	9.60	2.31	3.08
Ser	0.033-23.33	0.033	0.10	5.91	6.00	11.83	5.54	8.73	10.65	7.92	9.71	8.87
Thr	0.033-23.33	0.033	0.10	12.21	9.79	7.72	10.75	7.02	9.76	9.63	0.64	6.71
Asp	0.033-23.33	0.033	0.10	7.72	10.26	10.42	12.60	13.40	14.86	8.57	2.88	1.53
Met	0.033-23.33	0.033	0.10	10.79	14.12	6.53	10.52	10.75	11.96	6.44	2.51	1.55
Cys	0.033-23.33	0.033	0.10	12.98	8.92	6.75	10.44	6.25	10.88	1.61	8.07	0.41
Arg	0.033-23.33	0.033	0.10	11.96	10.18	7.51	11.66	14.33	14.79	3.00	4.78	8.91
Glu	0.033-23.33	0.033	0.10	10.67	10.50	13.10	12.60	12.15	9.95	3.23	2.72	0.63
Phe	0.033-23.33	0.033	0.10	9.20	13.83	5.36	8.01	6.23	8.57	5.36	7.97	0.20
Asn	1.19-23.33	1.19	3.59	11.03	7.48	14.66	8.28	6.57	8.85	7.81	8.85	0.94
Gln	0.033-23.33	0.033	0.10	12.41	5.36	8.14	5.90	10.87	13.68	9.89	5.94	3.90
His	1.19-23.33	0.033	0.10	7.18	5.45	6.36	11.59	9.39	6.30	1.45	7.83	2.48

Lys	0.033-23.33	0.033	0.10	7.78	8.58	12.01	13.97	13.69	13.60	6.05	5.77	0.96
Tyr	0.23-23.33	0.233	0.70	5.92	10.69	5.54	9.74	7.41	6.70	3.02	7.82	8.63
Trp	0.33-23.33	0.333	1.00	7.79	13.98	11.47	5.89	8.70	6.63	9.78	0.40	5.44
Ornithine	0.033-23.33	0.033	0.10	8.94	14.50	12.18	10.11	8.16	11.40	6.01	3.90	7.53
Dihydroxyphenylacetic acid	0.033-23.33	0.033	0.10	13.19	8.10	14.24	11.14	13.03	9.51	7.12	5.81	7.10
Tyramine	0.033-23.33	0.033	0.10	7.02	11.39	6.82	10.75	9.54	5.75	8.37	3.57	5.28
Dopamine	0.033-23.33	0.033	0.10	10.91	5.47	13.19	12.80	7.78	14.88	6.30	4.76	2.22
Tryptamine	0.033-23.33	0.033	0.10	7.78	8.58	12.01	13.97	13.69	13.60	6.05	5.77	0.96
Quinolinic acid	1.19-23.33	1.19	3.57	13.92	10.69	5.54	9.74	7.41	6.70	5.02	7.82	8.63
3-Hydroxyanthranilic acid	0.033-23.33	0.033	0.10	5.91	6.00	11.83	5.54	8.73	10.65	4.92	9.71	8.87
C6:0	33.33-333.33	33.33	100.00	2.52	7.17	6.51	7.77	3.88	9.07	2.47	3.71	8.76
C8:0	23.33-333.33	23.33	70.00	2.50	5.78	5.91	4.98	3.26	6.13	4.56	2.67	5.20
C10:0	10.00-333.33	10.00	30.00	7.62	7.58	3.53	7.29	5.83	2.41	7.53	4.14	7.58
C11:0	10.00-333.33	10.00	30.00	4.90	4.85	3.35	2.62	3.76	3.63	7.84	2.60	6.35
C12:0	10.00-333.33	10.00	30.00	6.79	7.27	2.95	4.54	2.35	2.62	4.37	5.73	5.73
C13:0	10.00-333.33	10.00	30.00	3.69	3.12	5.30	2.97	5.27	6.83	2.11	6.80	6.93
C14:1	10.00-333.33	10.00	30.00	2.51	2.81	6.56	3.99	4.75	6.44	5.52	3.04	4.09
C14:0	10.00-333.33	10.00	30.00	5.20	4.88	7.45	2.34	6.03	7.50	2.27	4.71	3.69
C15:1	10.00-333.33	10.00	30.00	4.87	6.02	5.86	5.22	5.35	2.26	5.26	4.29	5.37
C15:0	10.00-333.33	10.00	30.00	3.56	5.78	2.04	3.78	6.34	2.52	2.68	3.41	2.27
C16:1	10.00-333.33	10.00	30.00	5.95	4.72	7.02	3.19	7.39	7.14	5.06	2.95	2.71
C16:0	23.33-333.33	23.33	70.00	7.78	5.13	4.59	7.84	4.81	7.88	4.89	6.04	7.61
C17:1	10.00-333.33	10.00	30.00	2.52	6.17	2.51	7.77	3.88	7.07	2.47	3.71	2.76
C17:0	10.00-333.33	10.00	30.00	6.75	2.91	6.13	5.32	7.13	2.95	3.97	7.01	2.49
C18:3 T	10.00-333.33	10.00	30.00	2.71	7.73	2.01	7.78	2.47	3.10	5.12	2.01	6.21
C18:2 T	10.00-333.33	10.00	30.00	4.55	3.45	6.47	4.49	7.90	4.20	5.35	4.12	4.44
C18:1 T	10.00-333.33	10.00	30.00	3.36	7.54	7.72	6.30	3.45	3.49	4.76	3.41	4.57
C18:2	10.00-333.33	10.00	30.00	3.29	7.14	7.44	3.28	7.21	4.31	6.19	2.53	6.82
C18:1	10.00-333.33	10.00	30.00	5.36	7.07	7.39	2.08	4.85	3.76	6.16	2.82	2.06
C18:0	10.00-333.33	10.00	30.00	3.65	2.93	6.69	6.01	3.79	7.02	2.52	4.35	6.23
C20:4	100.00-333.33	100.00	300.00	6.16	4.35	5.78	5.87	3.56	3.65	7.34	4.34	7.01
C20:5	100.00-333.33	100.00	300.00	7.90	4.99	6.44	2.13	7.86	2.80	5.01	4.67	7.19
C20:3 T	33.33-333.33	33.33	100.00	6.49	7.37	6.83	3.33	3.90	6.97	3.86	7.55	2.38
C20:2	23.33-333.33	23.33	70.00	4.55	5.53	7.50	6.05	2.30	3.48	2.48	7.01	6.67
C20:1	100.00-333.33	100.00	300.00	4.19	6.79	7.68	4.26	7.94	5.92	2.49	5.04	6.16
C20:3	100.00-333.33	100.00	300.00	2.55	6.43	4.13	2.91	4.29	2.13	4.02	5.96	7.31

C20:0	10.00-333.33	10.00	30.00	2.27	3.75	4.69	3.49	7.53	6.89	3.61	4.64	7.84
C21:0	16.67-233.33	16.67	50.00	6.23	4.56	3.14	4.94	3.27	6.31	7.71	2.37	3.28
C22:6	100.00-333.33	100.00	300.00	7.78	5.35	2.57	4.91	4.48	4.01	2.09	3.98	5.56
C22:4	166.67-333.33	166.67	500.00	5.12	7.24	2.08	6.99	3.40	4.53	3.02	7.83	4.61
C22:5	166.67-333.33	166.67	500.00	4.77	5.22	5.01	5.76	5.60	4.60	6.28	2.69	2.52
C22:2	100.00-333.33	100.00	300.00	3.44	6.09	4.40	2.76	3.70	4.73	4.30	3.01	7.24
C22:1	100.00-333.33	100.00	300.00	5.20	4.71	3.76	6.53	3.05	6.13	6.79	4.28	5.62
C22:0	10.00-233.33	10.00	30.00	3.37	2.26	3.11	5.92	3.77	2.12	7.12	5.72	5.90
C23:0	16.67-233.33	16.67	50.00	2.30	4.27	5.02	6.35	5.08	3.04	7.95	2.80	5.49
C24:1	100.00-333.33	100.00	300.00	7.17	4.84	3.57	2.88	6.15	7.32	2.58	7.29	7.29
C24:0	100.00-333.33	100.00	300.00	3.34	6.16	7.60	3.23	4.24	5.95	5.11	2.85	5.05

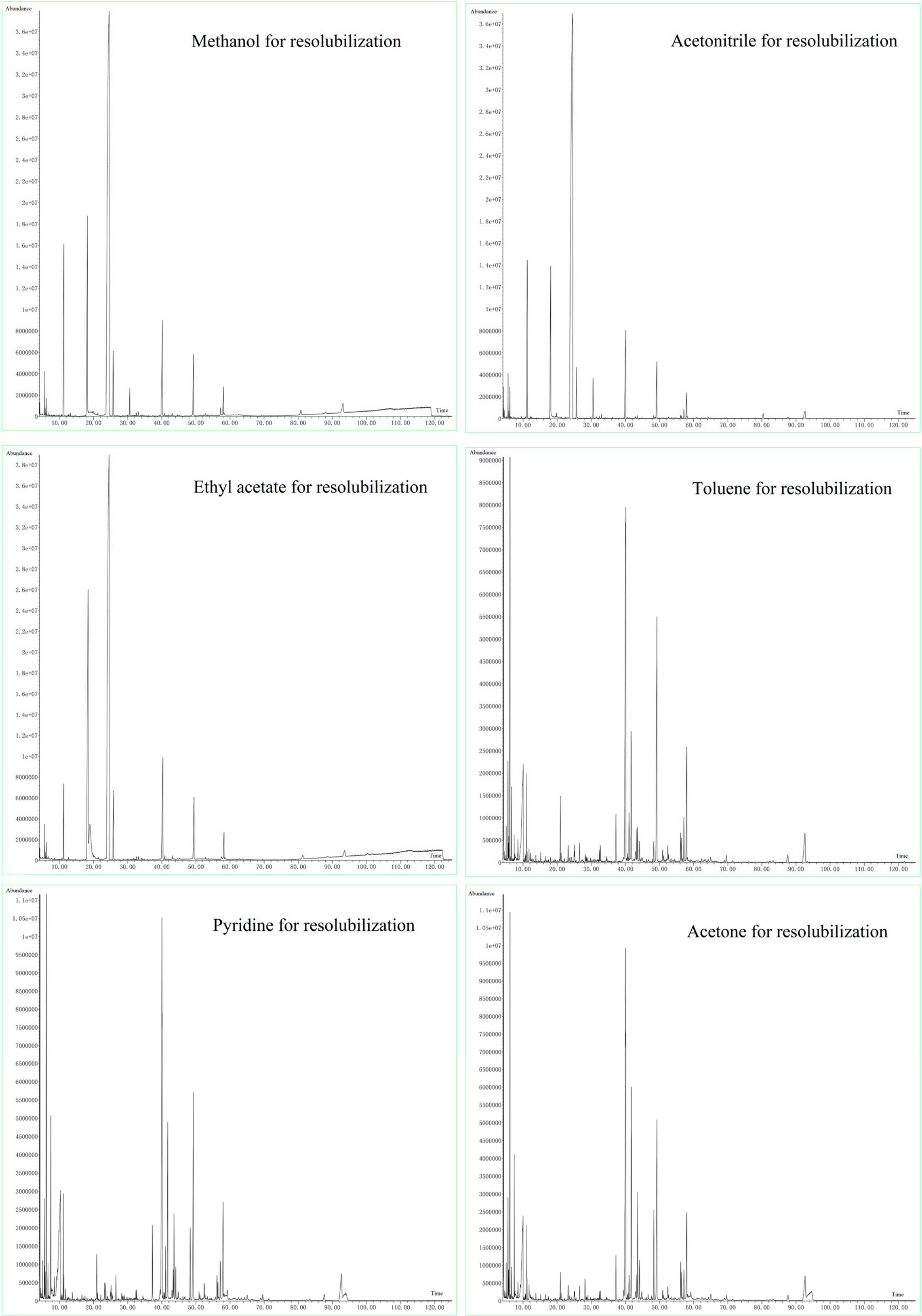

Figure S1 Comparison of the effect of different solvents on redissolution.

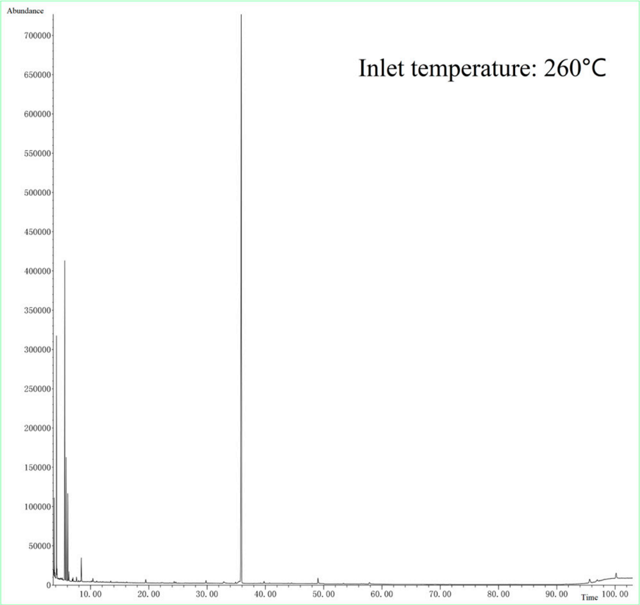
Abundance
Inlet temperature: 260°C
700000
650000
600000
550000
500000
450000
400000
350000
300000
250000
200000
150000
100000
50000
0
10.00
20.00
30.00
40.00
50.00
60.00
70.00
80.00
90.00
Time
100.00

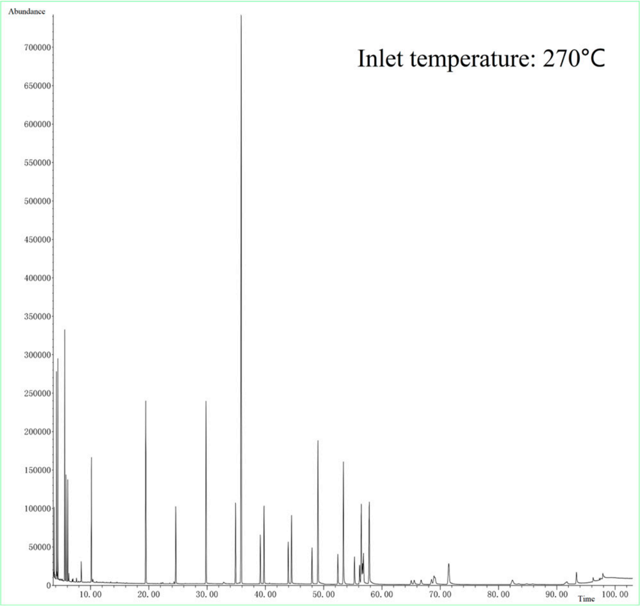
Abundance
Inlet temperature: 270°C
700000
650000
600000
550000
500000
450000
400000
350000
300000
250000
200000
150000
100000
50000
0
10.00
20.00
30.00
40.00
50.00
60.00
70.00
80.00
90.00
Time
100.00

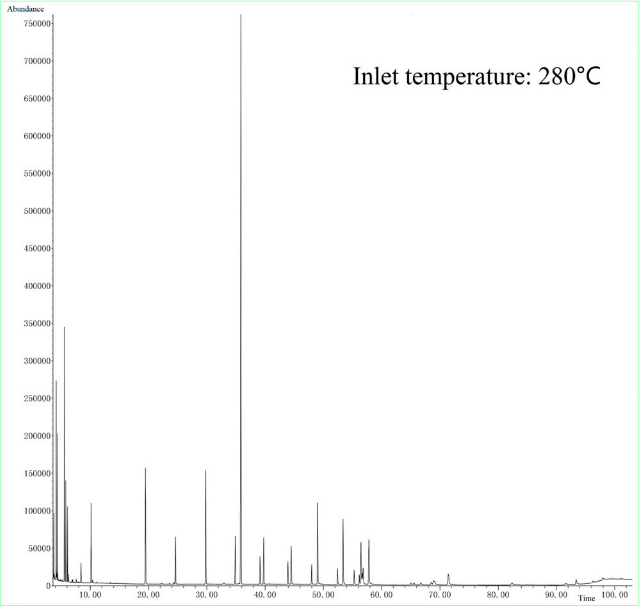
Abundance
Inlet temperature: 280°C
750000
700000
650000
600000
550000
500000
450000
400000
350000
300000
250000
200000
150000
100000
50000
0
10.00
20.00
30.00
40.00
50.00
60.00
70.00
80.00
90.00
Time
100.00

Figure S2 Comparison of different inlet temperatures.

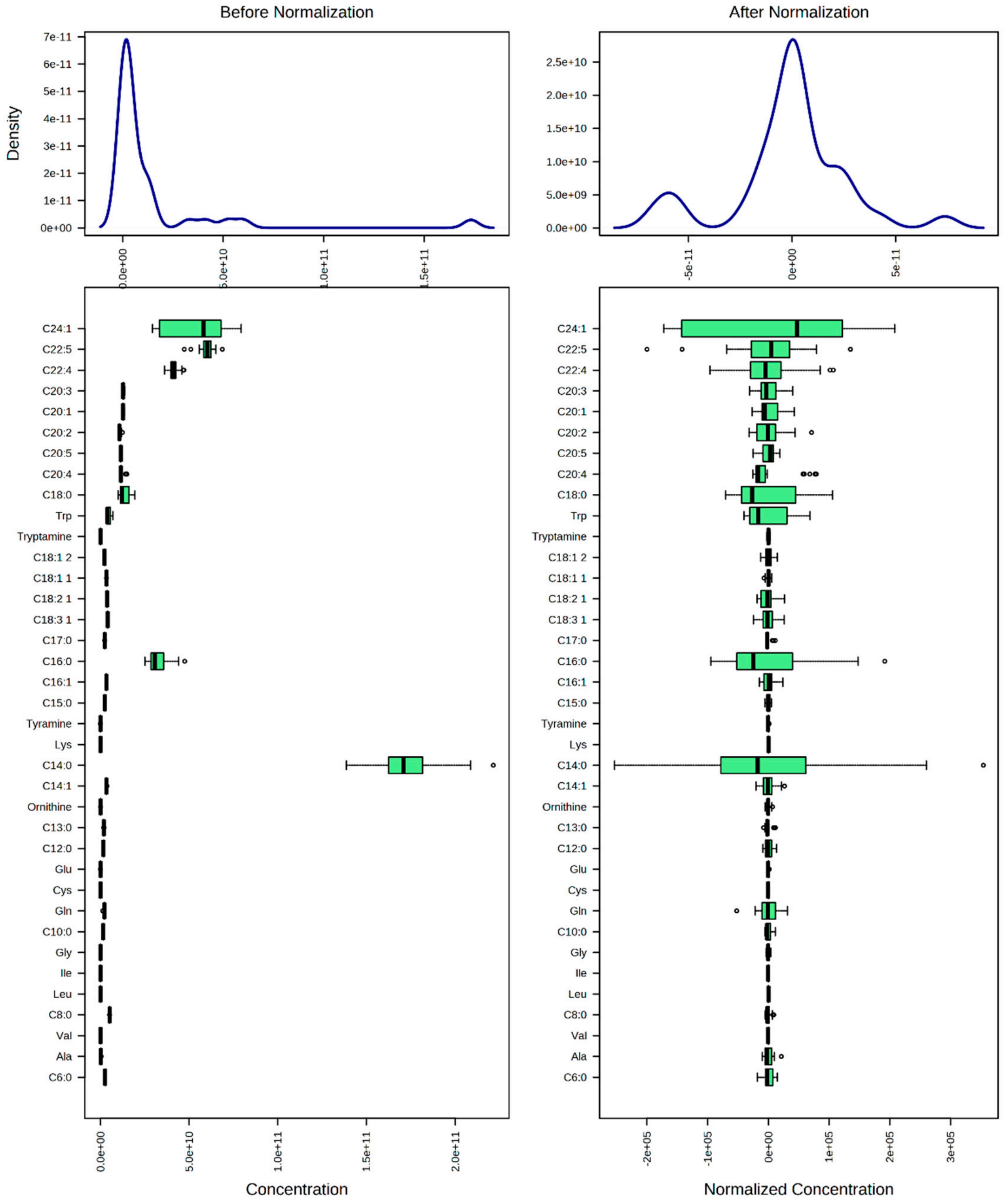

Figure S3 Data of targeted metabolism were compared before and after normalization.

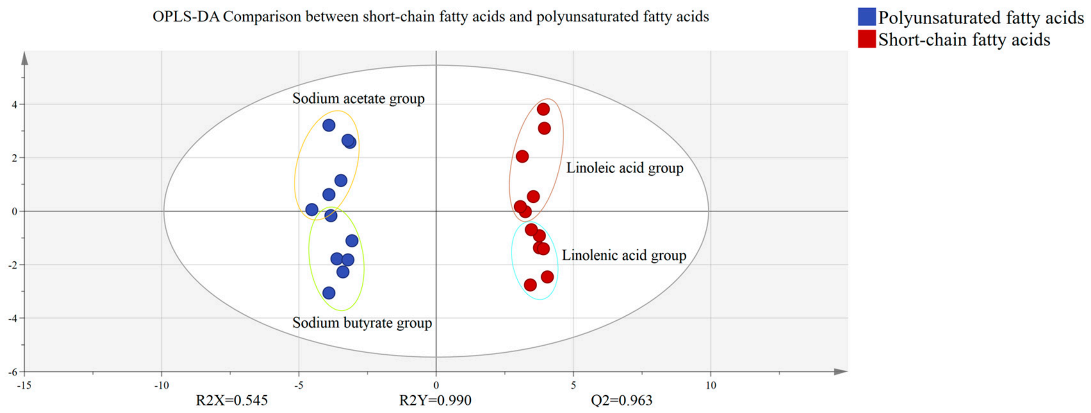

Figure S4 OPLS-DA plots between short-chain fatty acids and polyunsaturated fatty acids.
